# Day-to-day variability of [^68^Ga]Ga-PSMA-11 accumulation in primary prostate cancer: effects on tracer uptake and visual interpretation

**DOI:** 10.1186/s13550-020-00708-z

**Published:** 2020-10-30

**Authors:** Judith olde Heuvel, Berlinda J. de Wit-van der Veen, Maarten L. Donswijk, Cornelis H. Slump, Marcel P. M. Stokkel

**Affiliations:** 1 Department of Nuclear Medicine, Netherlands Cancer Institute-Antoni van Leeuwenhoek, Plesmanlaan 121, 1066 CX Amsterdam, The Netherlands; 2 Technical Medical Centre, University of Twente, Enschede, The Netherlands

**Keywords:** Repeatability, PSMA PET/CT, Primary prostate cancer, Test–retest, Tracer uptake

## Abstract

**Purpose:**

Prostate-specific membrane antigen (PSMA) agents, such as [^68^Ga]Ga-PSMA-11, have an unprecedented accuracy in staging prostate cancer (PCa) and detecting disease recurrence. PSMA PET/CT may also be used for response monitoring by displaying molecular changes, instead of morphological changes alone. However, there are still limited data available on the variability in biodistribution and intra-prostatic uptake of PSMA targeting radiotracers. Therefore, the aim of this study was to assess the repeatability of [^68^Ga]Ga-PSMA-11 uptake in primary PCa patients in a 4-week interval.

**Methods:**

Twenty-four primary PCa patients were prospectively included, who already were scheduled for [^68^Ga]Ga-PSMA-11 PET/CT scan on clinical indication (≥ cT3, Gleason score ≥ 7 or PSA ≥ 20 ng/mL). These patients received two [^68^Ga]Ga-PSMA-11 PET/CT scans with a 4-week interval. No treatment was started in between the scans. Semiquantitative measurements (SUL_max_, SUL_mean_, and SUL_peak_) were determined in the prostate tumor, normal tissues, and blood pool. The repeatability coefficient of every region was determined. All scans were visually analyzed by two nuclear medicine physicians.

**Results:**

Within-subject coefficient of variation of [^68^Ga]Ga-PSMA-11 uptake between the two scans was on average 10% in the prostate tumor, normal tissues (liver, kidney, parotid), and blood pool. The repeatability coefficient of the prostate tumor was 18% for SUL_peak_ and 22% for SUL_max_. Lesion uptake was visually different in 5 patients, though not clinically relevant.

**Conclusion:**

Results of test-retest [^68^Ga]Ga-PSMA-11 PET/CT scans in a 4-week interval show that [^68^Ga]Ga-PSMA-11 uptake is repeatable, with a clinical irrelevant variation in tumor and physiological distribution. Based on the presented repeatable uptake, [^68^Ga]Ga-PSMA-11 PET/CT scans can potentially be used for disease surveillance and therapy response monitoring. Changes in uptake larger than the RC are therefore likely to reflect actual biological changes in PSMA expression.

*Trial registration* NL8263 at Trialregister.nl retrospectively registered on 03-01-2020. https://www.trialregister.nl/trial/8263

## Introduction

Prostate cancer (PCa) is the second most common cancer amongst men in the world, as recorded in 2018 [1]. Molecular imaging of this malignancy either in primary or metastatic setting is presently dominated by the ligands directed to the prostate-specific membrane antigen (PSMA). This is a membrane-bound enzyme which is overexpressed in PCa cells compared to benign prostatic tissue by approximately 100- to 1000-fold [2, 3]. ^68^Gallium-labeled PSMA compounds, such as [^68^Ga]Ga-PSMA-11, is therefore considered a highly tumor-specific radiotracer for PCa. Since PSMA agents have an unprecedented accuracy in recurrent PCa, it has been rapidly adopted in the clinic over the last years [4, 5]. In staging of primary PCa, Hofman et al*.* recently published a prospective study, showing the higher diagnostic accuracy of [^68^Ga]Ga-PSMA-11 PET/CT in men with high-risk primary prostate cancer, as compared to conventional imaging (CT and bone scan) [6], which is as well supported by retrospective studies [7–9].

In many solid tumors, active surveillance and response monitoring with ^18^fluorine-fludeoxyglucose (2- [^18^F]FDG) PET/CT is quite common, and adopted in various guidelines [10]. Variations in [^18^F]FDG-accumulation can provide valuable information on the activity and efficacy of new cancer therapeutics. The Positron Emission Tomography Response Criteria in Solid Tumors (PERCIST) or European Organization for Research and Treatment of Cancer (EORTC) criteria are often used to quantify the response on therapy using [^18^F]FDG PET/CT. These criteria classify the disease status as ‘responder’, ‘progressed’, or ‘stable’ based on changes in the semiquantitative standard uptake values (SUV), corrected for lean body mass (SUL) [10]. However, [^18^F]FDG PET/CT is usually not suitable in PCa, as most tumors show limited FDG-accumulation, especially in hormone-sensitive setting.

Monitoring the response after therapy with [^68^Ga]Ga-PSMA-11 PET/CT may be helpful in PCa, yet this approach is not validated yet [11]. As with FDG-accumulation, the extent and intensity of the PSMA-uptake can be compared between scans to quantify response, and when deemed necessary adjust therapy. However, not many papers are published about response monitoring or active surveillance using [^68^Ga]Ga-PSMA-11 PET/CT [12]. One study by Gupta et al. [13] compared the functional criteria PERCIST 1.0 and EORTC in [^68^Ga]Ga-PSMA-11 PET/CT with the morphological criteria according to RECIST V1.1 in patients with metastatic PCa and biochemical progression. According to this study, molecular imaging criteria performed best in detecting progression based on changes of ≥ 25% SUV_mean_ (EORTC) or ≥ 30% SUL_peak_ (PERCIST) after hormone treatment [13]. However, the study does not describe if the biological variation in [^68^Ga]Ga-PSMA-11 uptake is comparable to that of [^18^F]FDG, and if the same cutoff values apply, while only changes that exceed the normal variability should be interpreted as treatment response or disease progression*.* Although two additional studies assessed this test–retest repeatability in metastatic prostate cancer using [^68^Ga]Ga-PSMA-11 [14] and [^18^F]DCFPyL [15], no studies in the primary setting are performed to this day, as far as we are aware of. Therefore, the aim of this study was to assess the day-to-day variability of [^68^Ga]Ga-PSMA-11 uptake and visual interpretation in patients with primary prostate cancer.

## Methods

### Patients

This prospective clinical study was performed at the Netherlands Cancer Institute (Amsterdam, the Netherlands). The study protocol was approved by the local Medical Ethics Committee (NL8263 at trialregister.nl), and all patients provided written informed consent. Men (≥ 18 years) with biopsy-proven PCa and a clinical indication to perform a [^68^Ga]Ga-PSMA-11 PET/CT scan (e.g., either ≥ cT3, Gleason score ≥ 7 or PSA ≥ 20 ng/mL) were eligible. Patients were excluded if no elevated [^68^Ga]Ga-PSMA-11 uptake was visible in the primary prostate tumor on the first scan, or when treatment was started in between the two scans.

### Study protocol

The first [^68^Ga]Ga-PSMA-11 PET/CT scan was performed on clinical indication based on the aforementioned criteria. Both PET/CT scans were performed according to the same local clinical protocol, consisting of adequate oral hydration before an intravenous bolus injection of 100 ± 10 MBq Glu-urea-Lys-(Ahx)-[^68^Ga]-HBED-CC ([^68^Ga]Ga-PSMA-11), which was radiolabeled in-house using a fully automated system (Scintomics GmbH, Fürstenfeldbruck, Germany). After an incubation time of 45 ± 5minutes, acquisitions were performed on a Vereos digital PET/CT system (Philips, Best, the Netherlands). Acquisition time was 3 min per bed position (min/bp) for the pelvic area and 2 min/bp toward base of skull. In the last phase of the study, clinical protocol changed to an administered activity of 150 ± 15 MBq [^68^Ga]Ga-PSMA-11 with acquisitions of 4.5 min/bp around the pelvis to improve image quality. CTs were acquired for attenuation correction and anatomical correlation. In general, no furosemide was given to the patients. The second scan was scheduled roughly 4 weeks later, for which deviations in administered activity within ± 10% and in time between injection and acquisition of within ± 5 min are aimed for.

### Image reconstruction and analysis

Data were reconstructed at 3 iterations, 8 subsets with Gaussian blurring (3 mm) and voxel size of 2 × 2 × 2 mm (matrix size 512 × 512). Semiquantitative measures were determined using either Osirix MD (Pixmeo SARL, Switzerland) or 3D Slicer (www.slicer.org). Spherical volumes-of-interest (VOI) was drawn to determine average uptake in the primary tumor (ø1.7 cm), normal tissues (i.e., right parotid gland ø2.5 cm, liver ø5cm, right kidney ø3.5 cm, fourth lumbar vertebra bone marrow ø2.5 cm), and blood pool activity in the abdominal aorta and ascending aorta (ø1.7 cm). Minimal VOI diameter of 1.7 cm was chosen to reduce partial volume effect. If present, [^68^Ga]Ga-PSMA-11 positive metastatic bone lesions or lymph nodes were included in the analysis. The blood pool was used as reference value [12, 16]*.* The standard uptake value corrected for lean body mass (SUL) was used for quantitative analyses [17, 18].

In the primary tumor, SUL_peak_ was defined as the 1cm^3^ with the highest activity concentration in the VOI. If multifocal lesions in the prostate were present, the SUL_peak_ of the hottest lesion was shown. The tumor-to-blood ratios (TBRs) were also determined, as they were found to best describe the tumor tracer uptake [19]. The relative difference between scan 1 and 2 was calculated for all indices.

### Visual assessment

Visual analysis was performed by two nuclear medicine physicians (MLD and MPMS) with experience in reading [^68^Ga]Ga-PSMA-11 PET/CT to assess any visual differences between the acquisitions. Both physicians were blinded to clinical parameters, possible other imaging and to which scan was made first and second. If visual differences were noted, the location of these differences was recorded, and scored as deviation in either bio-distribution or lesion uptake. Any disagreement between the physicians was settled in consensus.

### Statistical analysis

All statistical analyses were performed using SPSS 25 (IBM, Armonk, USA). The difference between tracer uptake time and injected activities of the two scans was assessed using paired *T*-tests. Repeatability was evaluated using different metrics, difference (*d*), relative difference (*D*), repeatability coefficient (RC), and within-subject coefficient of variation (wCV) [20, 21]. The wCV is the variance of the repeated measurements of individual subjects. The RC denotes the absolute difference between repeated measurements, which lies within the 95% confidence interval. The smaller these value, the better the repeatability

$$d={\rm SUL}_{2}-{\rm SUL}_{1}$$

$$D=\frac{{\rm SUL}_{2}-{\rm SUL}_{1}}{\stackrel{-}{\rm SUL}}\times 100\%$$

$${\text{wCV}}=\frac{\rm {Standard\;deviation} (D)}{\sqrt{2}}$$

$${\text{RC}}=1.96 \times {\text{wCV}}\times \sqrt{2}$$

## Results

### Patients and PET imaging

A total of twenty-four patients were included in this study. Two patients were excluded, as no elevated PSMA expression was observed in the prostate on the first scan. Patient demographics are shown in Table 1. The average injected activity was not statistically different between the two scans (99.8 MBq [range 81–113] and 103.8 MBq [range 96–110]; *p* = 0.06, for *n* = 20), as was the interval between radiotracer injection and scan (45.1 [range 38–57] and 46.1 min [range 41–64]; *p* = 0.42, for *n* = 22). The average time difference between administration and acquisition for the two scans was 4 min (0–19 min) and 7% (0–22%) difference in injected activity. Four patients violated the 10% difference in injected dose (22% and 28%) or 5 min deviation (9 and 19 min) in tracer incubation time between the two scans. In two patients, the new protocol was applied, with an injected activity at scan 1 of 136.7 and 146.9 MBq and at scan 2 130.8 and 139.6 MBq, respectively.

**Table 1:** Demographics of the patients included in this study. Data are shown as absolute value (percentage) or as mean (range)

		Number (%) or mean (range)
Age (years)		71 (53–81)
Gleason score		
	6	1 (5%)
	3 + 4 = 7	2 (9%)
	4 + 3 = 7	9 (41%)
	8	5 (23%)
	9 + 10	5 (23%)
iPSA (ng/mL)		
	< 10	11 (50%)
	10–20	8 (36%)
	> 20	2 (9%)
T		
	cT1c	9 (41%)
	cT2	7(32%)
	cT3	6 (27%)
*N*		
	*N*0	21 (95%)
	*N*1	1 (5%)
*M*		
	*M*0	21 (95%)
	*M*1	1 (5%)
Prostate volume (cc) on MRI		48.0 (22–88)

*iPSA* initial prostate-specific antigen

### *[*^*68*^*Ga]Ga-PSMA-11 uptake in normal tissue and blood pool*

The average SUL_max_ and SUL_mean_, the (relative) differences, wCV and RC of every organ are displayed in Table 2. In the blood pool, a relative mean difference in SUL of 1% (range − 29.2 to + 24.5%) in [^68^Ga]Ga-PSMA-11 uptake was observed. The SUL difference between the bladder (RC: 122%) and kidney (RC: 24%) is larger than in the rest of the organs. With a RC of 18%, the smallest difference was observed in the liver. The effects on RC due to protocol violation (*n* = 4 patients) and change in acquisition protocol (*n* = 2 patients) are displayed in Additional file 1: Figures 1 and 2. In one patient, the parotid gland was not included in the analysis, as quantification was not accurate due to head movement.

**Table 2:** SUL_max_, SUL_mean_, SUL_peak_ for normal tissue and the prostate tumor (mean ± range). Next the absolute difference and the relative difference between scan 1 and scan 2. The within-subject coefficient of variation (wCV) in %, the coefficient of repeatability (RC), both in % as in absolute SUL values

Organ	Metric	Average SUL	Difference in SUL scan 2–1	Relative difference (%)	wCV (%)	RC (SUL)	RC (%)
Parotid Gland	SUL_max_	15.1 ± 3.3 (10.1, 23.1)	0.5 ± 1.2 (− 1.4, 2.6)	4.1 ± 9.0 (− 9, 21)	11.3	2.3	17.0
	SUL_mean_	9.6 ± 1.9 (5.8,13.7)	0.2 ± 1.2 (− 1.9, 2.3)	3.9 ± 11.9 (− 15, 26)	8.4	2.3	23.4
Aortic arch	SUL_max_	1.8 ± 0.3 (1.0, 2.5)	0.0 ± 0.3 (− 0.6, 0.8)	− 0.2 ± 18.6 (− 31, 38)	13.2	0.6	36.5
	SUL_mean_	0.9 ± 0.1 (0.5, 1.2)	0.0 ± 0.1 (− 0.3, 0.1)	0.2 ± 12.2 (− 29, 25)	9.6	0.2	26.6
Liver	SUL_max_	7.2 ± 1.5 (5.0, 9.6)	− 0.5 ± 1.3 (− 5.5, 1.3)	− 7.1 ± 15.8 (− 63, 21)	6.5	1.4	31.1
	SUL_mean_	3.7 ± 0.8 (2.3, 5.5)	0.3 ± 0.4 (− 1.1, 0.6)	− 3.4 ± 9.2 (− 25, 13)	11.2	0.8	17.9
Kidney	SUL_max_	42.5 ± 6.9 (27.1, 56.7)	0.3 ± 4.5 (− 6.4, 9.4)	1.0 ± 11.3 (− 17, 29)	8.6	8.5	22.2
	SUL_mean_	19.5 ± 3.8 (11.6, 28.1)	− 0.5 ± 2.4 (− 5.2, 3.4)	− 2.4 ± 15.0 (− 25, 13)	8.0	4.7	23.9
A. Abdominalis	SUL_max_	1.9 ± 0.4 (1.2, 2.8)	0.0 ± 0.4 (− 0.6, 0.8)	2.6 ± 18.2 (− 28, 44)	12.9	0.7	35.6
	SUL_mean_	0.9 ± 0.2 (0.4, 1.1)	0.0 ± 0.1 (− 0.2, 0.3)	1.8 ± 14.3 (− 29, 35)	10.7	0.3	29.7
Bone	SUL_max_	1.5 ± 0.4 (0.9, 2.5)	0.1 ± 0.4 (− 0.7, 0.8)	3.1 ± 25.2 (− 48, 45)	17.8	0.8	49.4
	SUL_mean_	0.5 ± 0.2 (0.3, 0.9)	0.0 ± 0.1 (− 0.2, 0.1)	− 6.0 ± 14.0 (− 33, 17)	10.0	0.2	27.7
Bladder	SUL_max_	12.5 ± 8.9 (3.3, 43.7)	− 1.6 ± 8.9 (− 40.2, 12.4)	2.0 ± 58.1 (− 112, 113)	41.1	17.2	113.8
	SUL_mean_	7.7 ± 5.2 (1.9, 32.2)	− 0.3 ± 6.4 (− 7.7, 22.6)	9.2 ± 62.6 (− 108, 128)	43.9	12.3	121.8
Prostate tumor	SUL_max_	10.7 ± 7.3 (3.1, 32.3)	− 0.2 ± 1.1 (− 2.2, 2.5)	− 0.5 ± 11.2 (− 18, 21)	7.9	2.2	21.9
	SUL_peak_	6.2 ± 3.5 (2.6, 13.3)	0.0 ± 0.6 (− 1.2, 1.4)	1.2 ± 9.2 (− 15, 19)	6.5	1.1	18.1
TBR	SUL_max_	5.9 ± 4.2 (1.7, 17.8)	− 0.2 ± 1.2 (− 3.3, 1.9)	− 1.0 ± 19.7 (− 43, 42)	13.8	2.4	38.4
	SUL_peak_	5.4 ± 1.3 (2.1, 12.5)	0.2 ± 0.6 (− 1.0, 2.4)	5.7 ± 17.1 (− 19.5, 27.6)	9.9	1.6	27.5

Values are displayed as mean ± standard deviation (min, max). Average SUL of each site was calculated as mean over all subjects and all scans. Difference SUL and the relative difference are calculated as the group average of the difference

**Fig. 1:**
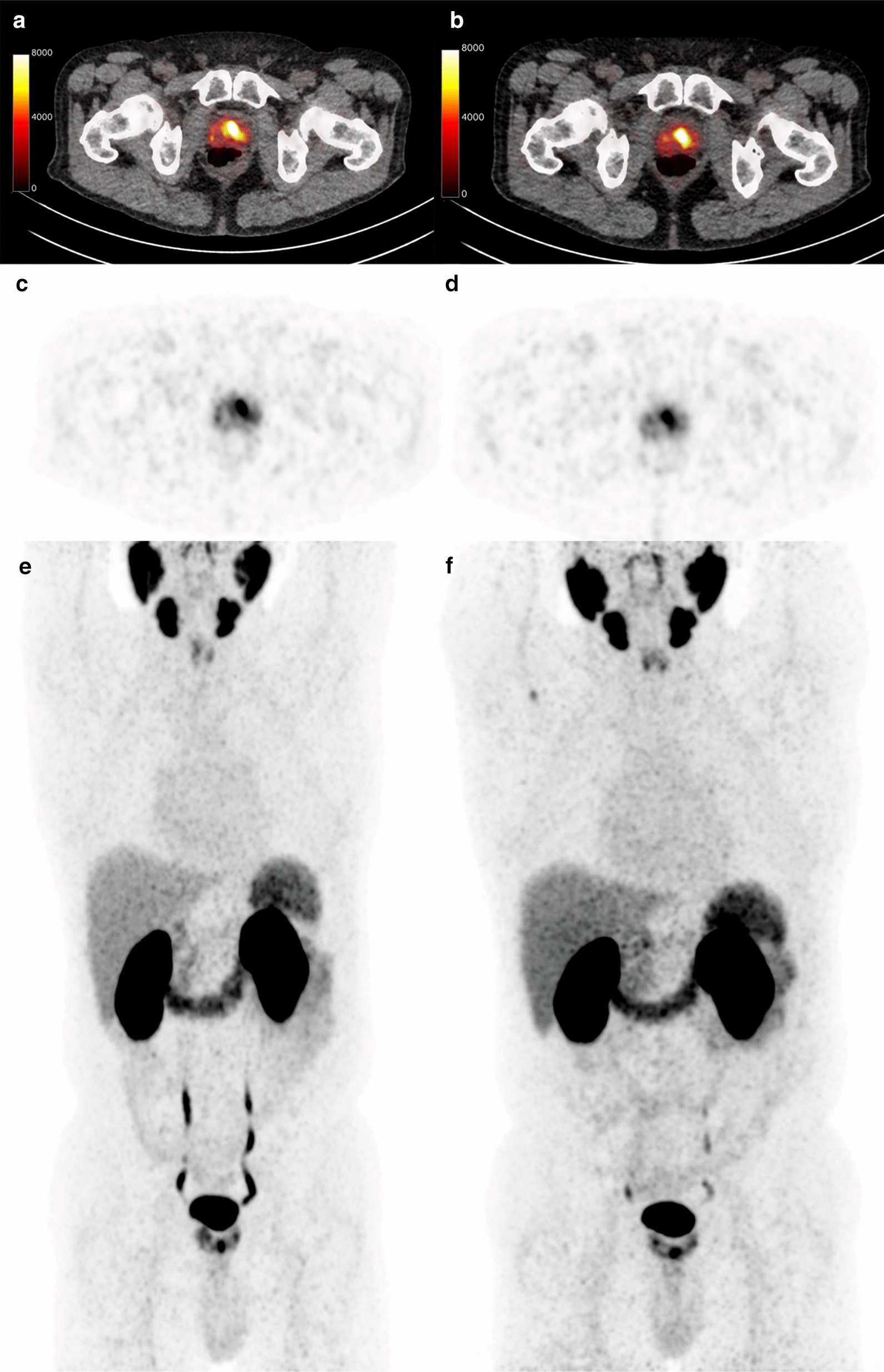
Example of a large difference between SUL_peak_ between scan 1 (**a**, **c**, **e**) and scan 2 (**b**, **d**, **f**), the difference in SUL_peak_ between both scans is 1.4

**Fig. 2:**
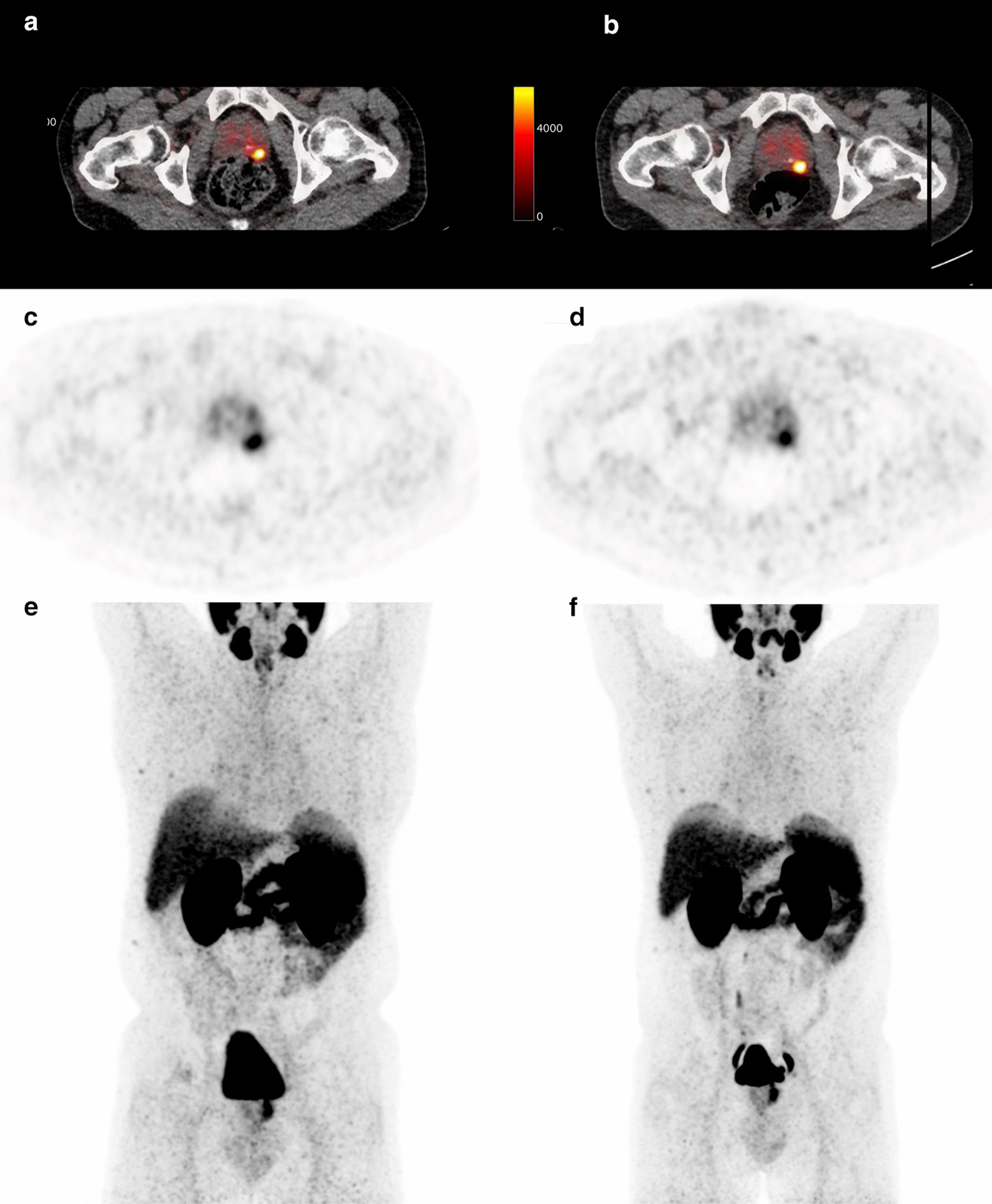
Example of a small difference between SUL_peak_ between scan 1 (**a**, **c**, **e**) and scan 2 (**b**, **d**, **f**), the difference in SUL_peak_ between both scans is 0.4

### *[*^*68*^*Ga]Ga-PSMA-11 uptake in primary tumor*

**Fig. 3:**
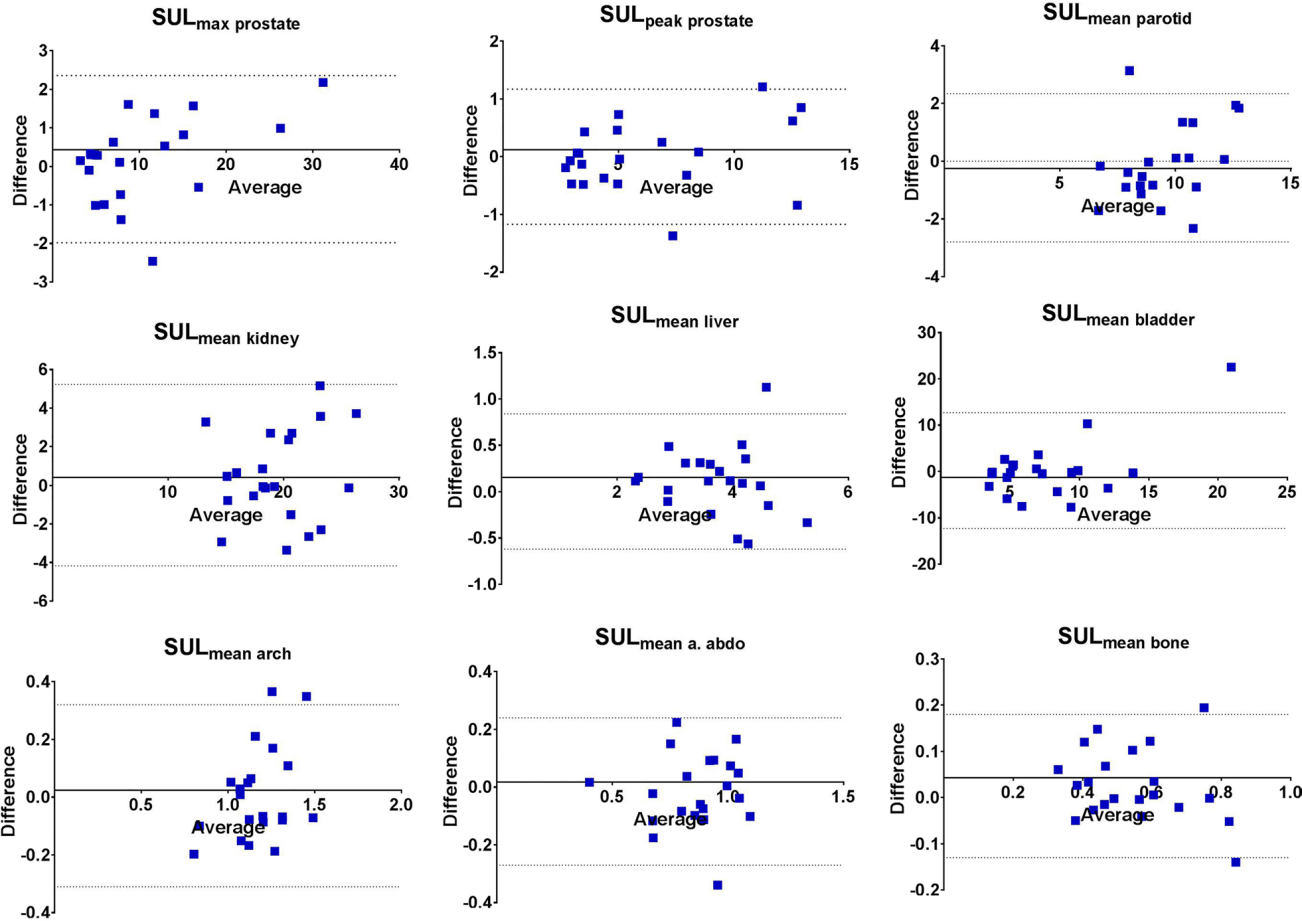
Bland–Altman plots of the absolute difference in SUL_max_, _peak_ of the prostate tumor, and SULmean of the parotid, liver, bladder, kidney, blood pool (abdominal aorta and ascending aorta), and bone. Horizontal lines represent the mean differences and the 95% confidence intervals of limits of agreement

On average, the relative mean difference in SUL_peak_ of the prostate tumor between the two scans was 1.2% (range − 14.5 to + 18.6%). The RC of SUL_max,_ and SUL_peak_ was 2.1 (21.9%) and 1.1 (18.1%), respectively. In general, the SUL_max_ is somewhat higher due to the larger impact of image noise, compared to SUL_peak_. Figure 3 shows the absolute differences between the two scans for both metrics against the average value of the two (i.e., Bland–Altman plot). Figures 1 and 2 show two examples of [^68^Ga]Ga-PSMA-11 PET/CTs with relative small and large variation in the SUL_peak_ of the primary tumor between the two scans. Note that though there are quantitative differences, the distribution pattern within the prostate is comparable in both examples. TBRs had a repeatability coefficient of 38.4% of SUL_max_, with an average difference of − 1% (range − 43 to + 41.5%). The effects on RC due to protocol violation (*n* = 4 patients) and change in acquisition protocol (*n* = 2 patients) are displayed in Additional file 1: Figures 1 and 2.

Figure 3 shows the Bland–Altman plots of the primary tumor, normal tissue and the blood pool. These plots show no clear association, in other words, that the repeatability is equally robust across a wide SUL range in normal tissue and in the prostate.

### Visual assessment

Visually, there was no difference between the two scans in 17 of the 22 cases with respect to the detectability and extent of the lesions. In three patients, visual differences between the two scans were noted with regard to the primary prostate lesion. In two other, in these particular cases, the visual differences did not have any impact on the clinical staging and subsequent treatment plan. With regard to the biodistribution, no visual differences in radiotracer accumulation were observed except for differences in bladder and urinary tract activity in six patients.

## Discussion

To our knowledge, repeatability of [^68^Ga]Ga-PSMA-11 PET/CT scans in the primary PCa setting has not been investigated before. This information is essential to perform response monitoring based on PSMA PET/CT. Therefore, the aim of this study was to assess the repeatability of [^68^Ga]Ga-PSMA-11 PET/CT scan in primary PCa patients in a 4-week interval.

The repeatability coefficient of SUL_peak_ in the prostate tumor was 18.1%, suggesting that below this value, the absolute difference between two scans in one patient (under the same circumstances) should fall within 95% probability. If the relative difference in SUL_peak_ is larger than ± 18%, then the difference is more likely to be explained by true changes and then by measurement errors. The absolute RC in the prostate tumor is probably less useful than the percentage, as there is a broad range in uptake between patients. The SUL_mean_ of normal tissue has a RC of 23.8% on average. In general, the RC of the SUL_max_ is higher than the RC of SUL_mean_, since SUL_max_ is more susceptible to noa. The RC of the SUL_mean_ of the blood pool was 26%. However, due to the very low radiotracer activity in the bone and blood pool, small absolute differences in SUL_mean_ can already lead to a large percentage difference. Thus, in these organs, it is more informative to look at the repeatability coefficient of the absolute difference, which is 0.2 for the blood pool and 0.2 for bone.

The visual differences between the two scans were not considered clinically relevant by the expert readers, and were predominantly related to a variable urinary excretion or variances in noise levels. Particularly, in three patients the activity in the prostate was noisy; therefore, the primary tumor was difficult to distinguish from the background, probably explaining the initial inter-observer differences in visual assessment. Next, in one of the patients, the visual appearance of a lymph node was different, which might have been caused by a larger (22%) deviation in the injected dose between the two scan points in that particular case. Still, in this patient the tumor and other organs were not visually different.

The current study had quite a similar setup as previous studies described in the literature, but there are some relevant differences [14, 15]. Previous studies looked at metastatic patients or [^18^F]DCFPyL instead of [^68^Ga]Ga-PSMA-11. Given the similar bio-distribution, findings for [^68^Ga]Ga-PSMA-11 may probably be generalized to [^18^F]DCFPyL [22]. Noteworthy is that [^18^F]PSMA-1007 has a distinctly different bio-distribution than the latter radiotracers [23]. Also, most other studies quantified uptake with body-weight-corrected SUV instead of SUL, yet the RC of SUL_max_ and SUV_max_ within the same patient in a test–retest setting is comparable. The RC of SUL_max_ of the prostate lesion in our study was 21.9%. Pollard et al*.* reported 30.3% and Jansen et al*.* 31.0% SUV_max_. The wCV of the prostate tumor in our study is 7.9% for SUL_max_, compared to 10.9% for SUV_max_ reported in the study by Pollard et al. [24]. These studies investigated patients with metastatic lesions as opposed to the patients with primary prostate cancer in our study. Metastatic lesions have a larger variation in PSMA receptor expression and general expansion of disease between patients than primary PCa tumors, thus possibly resulting in a higher RC than ours. When comparing our TBR findings to Jansen et al*.* [15], the RC is within the same range (38.4% vs 37.3%). The RC of the SUL is smaller than the RC of the TBR (21.9% vs 38.4% SUL_max_ vs TBR_max_), probably because TBR adds the SUL variation both the blood pool and the tumor. This was found by Jansen et al*.* as well [15]. In prior studies, a shorter window of not more than 7 days for repeatability was used. Due to the fact that prostate cancer is generally an indolent cancer, a 4-week interval was considered reliable, as shown in the present study.

In general, the SUL_max_ values of normal tissues found in the literature for PSMA PET/CT scans are comparable to ours [16, 25–27]. The differences between the two scans are largest in the ureter and bladder, which can be explained by variations in urine volume and radiotracer activity in the bladder. Though patients were not allowed to receive furosemide according to study protocol study, two patients had a protocol violation and received furosemide before one acquisition, resulting in a clear difference in bladder radiotracer activity (i.e., 2.1 vs 9.6 SUL_mean_). The differences between two scans are lowest in the liver, concordant to the findings of Li et al*.* [28]. The uptake variation of the parotid is on average repeatable (23.4%), although there is a large range, and comparable to the results of Pollard et al. (26.5%). However, comparing the SUL_mean_ to [^18^F]DCFPyL found by Li et al. [28], the parotid and kidney SUL_mean_ were lower than our findings. Still SUL_mean_ of the liver was equal.

The repeatability of [^68^Ga]Ga-PSMA-11 uptake in prostatic lesions is not entirely comparable to previous findings of [^18^F]FDG uptake in malignancies [21]. However, [^18^F]FDG scans do require a more concise preparation and patients’ adherence to the protocol [29]. In the day-to-day clinical setting, patients are likely to have variation in, for instance, blood glucose levels or physical activity, thus directly resulting in a less reproducible [^18^F]FDG uptake. The advantage of [^68^Ga]Ga-PSMA-11 PET/CT is that signal intensity depends primarily on the number of PSMA-expressing cells and expression density per cell [30]. In contrast to [^18^F]FDG, which accumulates to some extent in most tissues in the body, PSMA-ligands tend to accumulate only in specific tissues. Sahakyan et al. [31] found that variability in the liver and kidneys can be caused by intrapatient factors (i.e., time of day, recent meals, hydration status), and that interpatient factors (i.e., weight, height, body composition, medical comorbidities) can influence uptake in the salivary glands. Nonetheless, these influences were described in patients who underwent therapy, so it is difficult to distinguish between therapeutic effects and day-to-day physiological variations.

All these characteristics aid in repeatable quantitative [^68^Ga]Ga-PSMA-11 uptake in PCa lesions, and so it should allow for stable follow-up monitoring of the disease. A change in PSMA uptake in the tumor more than 18% as mentioned before may indicate either disease progression or treatment response. In a preclinical setting, PSMA expression is already used for response monitoring in taxane-based chemotherapy [32]. Note that careful image interpretation is needed when describing an increase or decrease in PSMA uptake [33]. Androgen deprivation therapy can influence the PSMA expression, where up- or downregulation is not unambiguously affected by type and duration of medication [34–36]. If PSMA PET/CT is used for response monitoring of radionuclide therapy, the tumor sink effect cannot be neglected [37].

### Limitations

Our study has some limitations that need some further elaborations. First, there was an alteration in the clinical imaging protocol while performing this study. The prescribed activity of [^68^Ga]Ga-PSMA-11 was increased from 100 MBq (*n* = 20) to 150 MBq (*n* = 2) in order to improve the image quality of PET/CT images in our institute. Though the effects on the SUL_mean_ and SUL_peak_ RC-values are limited (Additional file 1: Figure 1), the effect is somewhat larger in SUL_max_ measurements, as these are more susceptible to noise. Other aspects of the protocol like tracer uptake time, use of furosemide, and reconstruction settings were not altered. Although stringent protocol adherence was aimed for, four patients violated the ± 10% variation in administered dose and ± 5 min variation in uptake time. As this was not an exclusion criterion and might also occur in clinical practice as well, we decided to include these patients. This, however, did result in an increased repeatability compared to the use of a clean dataset without these violations (Additional file 1: Figure 2). Still, if centers want to perform response assessment with PSMA-PET/CT, it remains important to adhere to the protocol.

For image processing, VOIs were used instead of segmenting the entire organs to provide SUL_mean_ values. Still, Li et al. [27] found that there was no significant difference in SUL_mean_ of an entire segmented liver and a VOI with a 3 cm in diameter. In addition, images were not registered to each other before segmentation, and the VOIs were drawn by one person based on visual concordance of the location in the two PET/CTs. We believe that registration is not commonly used in clinical practice, as it might induce registration errors, and that the chosen segmentation approach mimics our clinical routine for response monitoring.

Next, we did not report on SUL_mean_ repeatability of the prostate tumor, as threshold-based segmentations using, for instance, 45% of SUL_max_ proved not an appropriate method, especially for tumors with low SUL_max_. In these patients, almost the entire prostate was segmented with this threshold, thus not representing the actual prostate tumor. To our knowledge, no standardized methods are yet published to accurately define the prostate lesion volume based on PSMA PET/CT. Since SUL_max_ is more sensitive to image noise, we chose to obtain the SUL_peak_ for prostate lesions.

## Conclusion

[^68^Ga]Ga-PSMA-11 PET/CT scans are repeatable in primary prostate cancer patients, with a repeatability coefficient of 18% SUL_peak_ in the primary prostate lesions within a 4-week time period. Variations found in the current study for normal tissues (liver, parotid) were comparable to those previously found in the metastatic setting. Based on these results, [^68^Ga]Ga-PSMA-11 PET/CT scans may be used for accurate surveillance and therapy response monitoring. Still, repeatability and distribution is different from [^18^F]FDG-PET/CT, indicating that EORTC- or PERCIST-criteria for solid tumors may not be suitable in [^68^Ga]Ga-PSMA-11 PET/CT.

## Supplementary information

**Additional file 1**. Two figures on the influence of the new protocol and protocol violations on the RC_mean_ and RC_max_, compared to the regular protocol.

## Data Availability

The datasets used and/or analyzed during the current study are available from the corresponding author on reasonable request.

## Abbreviations

BW: Body weight
CT: Computed tomography
*d*: Difference
*D*: Relative difference
EORTC: European organization for research and treatment of cancer
FDG: Fludeoxyglucose
*H*: Height
PCa: Prostate cancer
PERCIST: Positron emission tomography response criteria in solid tumors
PET: Positron emission tomography
PSA: Prostate-specific antigen
PSMA: Prostate-specific membrane antigen
RC: Repeatability coefficient
SUL: SUV corrected for lean body mass
SUV: Standard uptake value
TBR: Tumor‐to‐blood ratio
wCV: Within-subject coefficient of variation
