# Supplementary material for: Day-to-day variability of [68Ga]Ga-PSMA-11 accumulation in primary prostate cancer: effects on tracer uptake and visual interpretation
Source: EJNMMI Res. 2020 Oct 30;10:132. doi: 10.1186/s13550-020-00708-z (PMC7596127; doi:10.1186/s13550-020-00708-z)
Supplement: Supplementary file 1 — Additional file 1. Two figures on the influence of the new protocol and protocol violations on the RCmean and RCmax, compared to the regular protocol. [file 13550_2020_708_MOESM1_ESM.pdf]

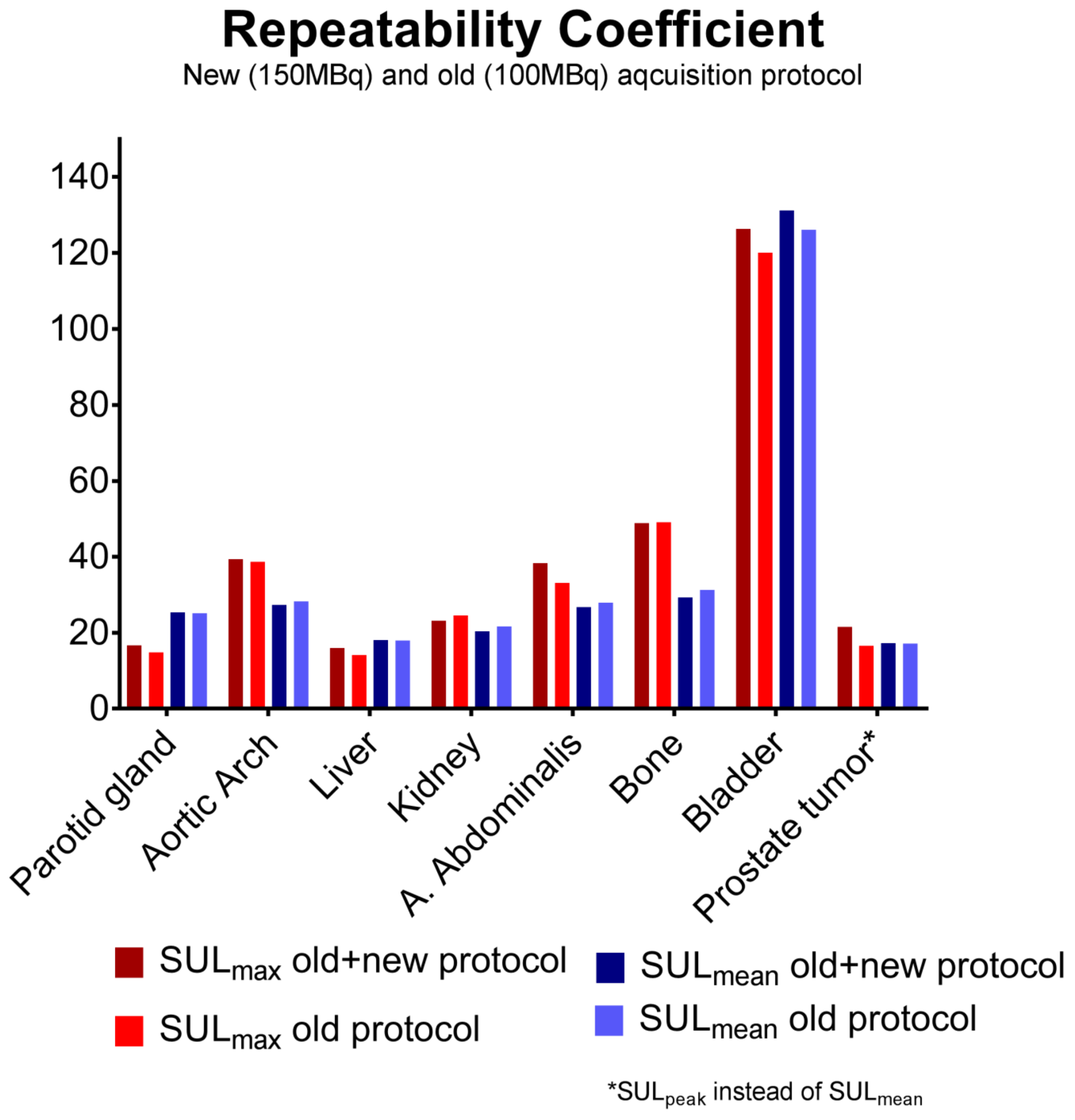

Supplementary Figure 1: Influence of the new protocol (150MBq & 4.5min/bp) on the RC_{mean} and RC_{max}, as compared to the old protocol (without protocol violations). Note that the value of the prostate is displayed as SUL_{peak} instead of SUL_{mean}.

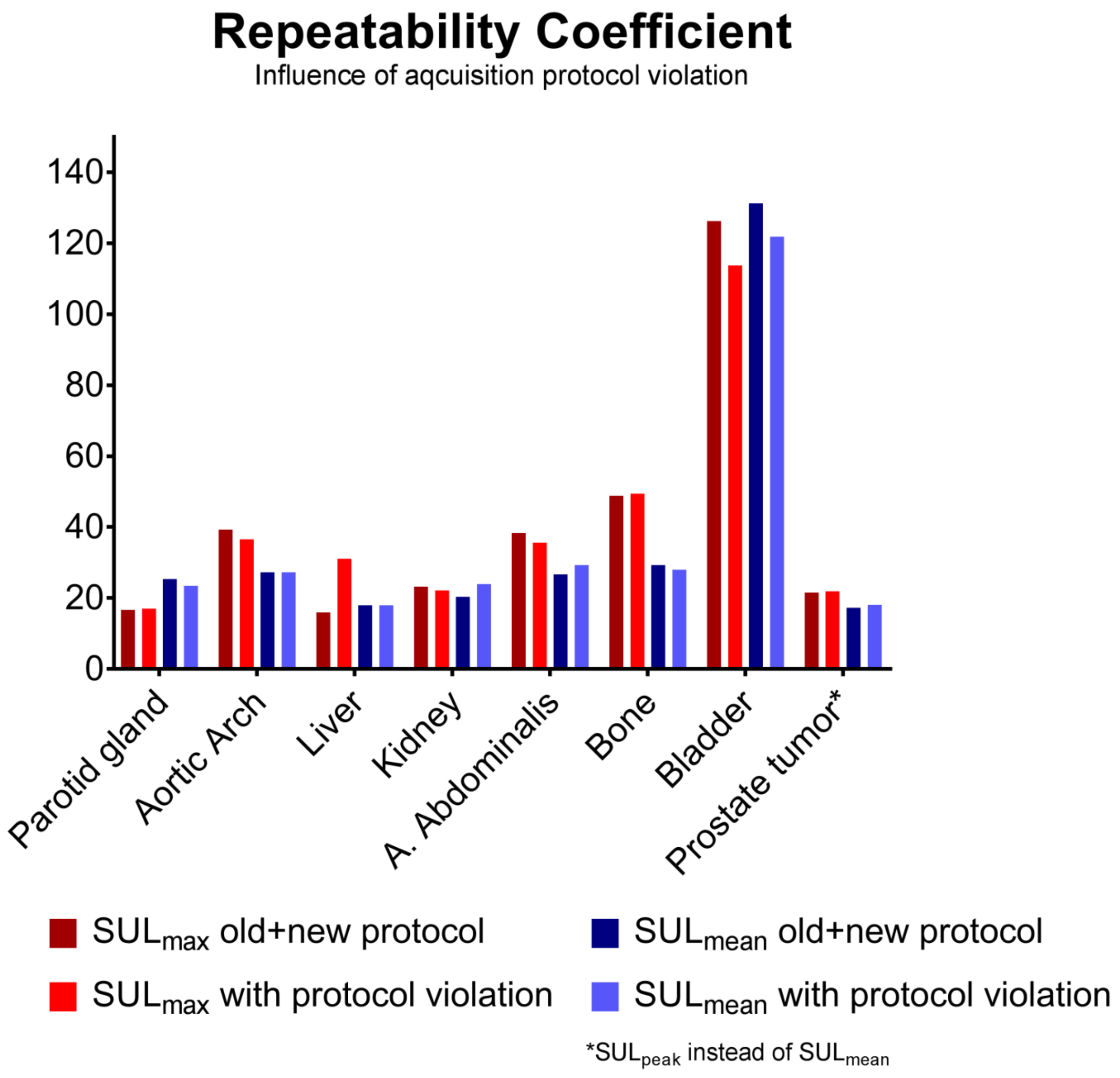

Supplementary Figure 2: The influence of the protocol violations (>10% difference in administered dose and >5min difference in tracer uptake time) on the RC_{mean} and RC_{max} were investigated by comparing the RC of the old and new protocol combined to the RC of the old+new protocol with the protocol violations included. Note that the value of the prostate is displayed as SUL_{peak} instead of SUL_{mean}. Note that the large difference in the SUL_{max} of the liver is caused by 1 patient, which difference between the scans is very large due to a noisy scan, the RC without this outlier is 18%, more close to the RC without protocol violation (RC 16%) .
